# Childhood Transverse Myelitis with Atypical Fever Presentation: A Case Report

**DOI:** 10.7759/cureus.64218

**Published:** 2024-07-10

**Authors:** Han Grezenko, Kimberly Kanemitsu, Khush Bakhat Khalid, Fnu Payal, Fnu Versha, Aakash Kumar, Shehryar Rehman

**Affiliations:** 1 Medicine and Surgery, Guangxi Medical University, Nanning, CHN; 2 Translational Neuroscience, Barrow Neurological Institute, Phoenix, USA; 3 Clinical Department, Windsor University School of Medicine, Chicago, USA; 4 Pediatric Medicine, Children Hospital and Institute of Child Health, Multan, PAK; 5 Medicine and Surgery, Quaid e Azam medical college, Bahawalpur, PAK; 6 Medicine, Shaheed Mohtarma Benazir Bhutto Medical University, Larkana, PAK; 7 Internal Medicine, People Medical University of Health and Sciences, Karachi, PAK; 8 Internal Medicine, Liaquat University of Medical and Health Sciences, Jamshoro, PAK; 9 Internal Medicine, Al Assad University Hospital, Damascus, SYR

**Keywords:** pediatrics, plasmapheresis, inflammatory condition, pediatric rare diseases, acute cervical transverse myelitis

## Abstract

Transverse myelitis (TM) is a rare inflammatory spinal cord disorder, particularly uncommon in children. It is characterized by symptoms such as motor weakness, sensory disturbances, and autonomic dysfunction. This report describes a 10-year-old male presenting with bilateral lower limb weakness, urinary and fecal incontinence, and high-grade fever. Initial treatment at a local hospital with corticosteroids and antibiotics did not yield significant improvements, prompting advanced care at a tertiary facility. A magnetic resonance imaging (MRI) confirmed a longitudinally extensive TM lesion. Subsequent management with plasmapheresis led to satisfactory clinical improvement. This case highlights the importance of early consideration of TM in pediatric patients with acute neurological deficits and supports the use of aggressive therapeutic strategies for better outcomes.

## Introduction

Pediatric transverse myelitis (TM) is a rare but serious inflammatory disorder that impacts the spinal cord, manifesting with a sudden onset of symptoms such as motor weakness, sensory disturbances, and autonomic dysfunction. TM results from inflammation, which can lead to demyelination and axonal injury within the spinal cord. Common etiologies include post-infectious processes, autoimmune conditions, and, less frequently, direct infections, vascular disorders, or paraneoplastic syndromes. In many cases, however, the cause remains idiopathic [1].

Clinically, pediatric TM often presents with a rapid progression of bilateral lower limb weakness, sensory loss, and autonomic dysfunction such as urinary and fecal incontinence. Pain, particularly back pain, and a band-like sensation around the trunk are also frequently reported. The severity and extent of symptoms can vary, with some children experiencing complete paralysis below the level of the lesion, while others may have milder motor and sensory deficits [2].

TM occurs infrequently, with an incidence of 1.76 per 1,000,000 in the pediatric population aged 19 years and younger. The typical age of onset for idiopathic acute TM in children is approximately 9.5 years, with cases ranging from as young as 0.5 years to 16.9 years old. The condition also exhibits a variable gender distribution, where the female-to-male ratio in children under 12 years is approximately 1.2:1, increasing to 2.8:1 in those aged 12 and older [2].

This case report aims to present a comprehensive account of the clinical presentation, diagnostic process, treatment strategies, and outcomes of a rare case of TM in a pediatric patient. Doing so aims to enrich the existing medical literature and enhance the understanding of this complex condition within the pediatric population. This report provides valuable insights into the challenges and considerations of managing pediatric TM, contributing to better clinical practices and outcomes for this vulnerable group.

## Case presentation

A 10-year-old previously healthy male presented with sudden-onset bilateral lower limb weakness and sensory loss after playing cricket and taking a bath. This was accompanied by high-grade fever and urinary and fecal incontinence, including bed-wetting. The timeline of symptom progression began with the sudden onset of bilateral lower limb weakness and sensory loss on Day 1, followed by the development of urinary and fecal incontinence on Day 2. On Day 3, the child received initial treatment at a local hospital, which included antibiotics and antipyretics, but a definitive diagnosis could not be established due to limited testing capabilities. By Day 5, the lack of improvement prompted a referral to a tertiary care center.

The patient’s medical history was notable for complete polio vaccination, with no familial history of neurological disorders. Despite experiencing a loss of appetite and thirst, there was no significant weight loss. Upon examination at the tertiary care center, the child was pallor but fully conscious with a Glasgow Coma Scale of 15/15. He demonstrated bilateral lower limb weakness, was unable to stand or walk, and had disturbed sensory perception in the lower limbs, though all cranial nerves and cerebellar functions were intact. Initial investigations revealed mild anemia, elevated eosinophils, slightly low potassium levels, and mild elevation in erythrocyte sedimentation rate (ESR) and C-reactive protein (CRP), suggestive of an inflammatory process. The drug history included the initial treatment at the local hospital, which comprised unspecified antibiotics. At the tertiary care center, the patient was administered high-dose intravenous (IV) corticosteroids and vancomycin as a precautionary measure against potential bacterial superinfections.

During the initial assessment, several differential diagnoses were considered, including acute flaccid myelitis, Guillain-Barré syndrome, and spinal cord compression due to various etiologies such as tumors or abscesses. Infectious causes, including viral myelitis and post-infectious autoimmune reactions, were also considered, although blood cultures were negative. Neuromyelitis optica spectrum disorder (NMOSD) was another differential, characterized by longitudinally extensive TM.

The baseline laboratory results supported the suspected diagnosis of TM, indicating an underlying inflammatory process and potential nutritional deficits. The results included mild anemia (hemoglobin: 9.1 g/dL), elevated eosinophils (12.2%), slightly low potassium levels (3.19 mmol/L), and mild elevation in ESR (20 mm/hr) and CRP (3 mg/L). Baseline laboratory results are given in Table 1.

**Table 1:** Baseline laboratory results. INR: international normalized ratio; APTT: activated partial thromboplastin time; WBC: white blood cells; RBC: red blood cells; HCT: Hematocrit; MCV: mean corpuscular volume; MCH: mean corpuscular hemoglobin; MCHC: mean corpuscular hemoglobin concentration; ALT: alanine transaminase; AST: aspartate aminotransferase; ESR: erythrocyte sedimentation rate; CRP: C-reactive protein

Test Category	Test	Result	Reference Range	Notes
Coagulation profile	Prothrombin time	12 s	10-14 s	Within normal limits
	INR	1.0	0.9-1.3	
	APTT	32 s	Up to 31 s	Slightly elevated
Hemogram	WBC count	7.23 x 10^9^/L	4-11 x 10^9^/L	Normal
	RBC count	3.77 x 10^12^/L	3.8-5.2 x 10^12^/L	Slightly low
	Hemoglobin	9.1 g/dL	13-18 g/dL	Indicates anemia
	Hematocrit (HCT)	28.9%	35-46%	Low, consistent with anemia
	MCV	77.9 fl	77-95 fl	Low normal
	MCH	24.4 pg	26-32 pg	Low, indicates hypochromia
	MCHC	31.8 g/dL	32-36 g/dL	Low normal
	Eosinophils	12.2%	1-6%	Elevated, suggests allergy or infection
Renal function tests	Urea	25 mg/dL	10-50 mg/dL	Normal
	Serum creatinine	0.23 mg/dL	0.5-0.9 mg/dL	Low
Liver function tests	ALT	39.1 U/L	Up to 40 U/L	Normal
	AST	28.9 U/L	Up to 40 U/L	Normal
Serum electrolytes	Potassium	3.19 mmol/L	3.5-5 mmol/L	Slightly low
Inflammatory markers	ESR	20 mm/h	0-25 mm/h	Mildly elevated
	CRP	3 mg/L	<5 mg/L	Normal
Urine examination	Protein	Trace	<150 mg/d	Non-significant
	Specific gravity	1.015	1.005-1.030	Normal
	pH	8	4.6-8.0	Normal

These results support the suspected diagnosis of TM, indicating an underlying inflammatory process and potential nutritional deficits. Further diagnostic evaluations, including micturating cystourethrogram (MCU) and magnetic resonance imaging (MRI), were conducted to explore these abnormalities and assess for structural or inflammatory causes.

The MCU findings consisted of two parts. The plain film revealed multiple gas-filled loops in the abdomen and pelvis, which obscured the underlying bony and soft tissue structures; however, there were no skeletal abnormalities, soft tissue abnormalities, abnormal calcifications, spina bifida, or hip joint abnormalities, and bone density was normal. The post-contrast images indicated a slightly thick-walled urinary bladder with mild bladder neck hypertrophy but no masses, diverticula, filling defects, or sacculation. There was no reflux of contrast into the ureters or kidneys, and the post-void residual volume was minimal. These findings suggest mild cystitis but no significant urological abnormalities.

A blood culture was performed to investigate potential infectious causes of the patient’s symptoms. The results were negative, showing no bacterial growth, which helps to exclude a bacterial infection as the underlying cause of the patient’s clinical presentation.

**Figure 1:**
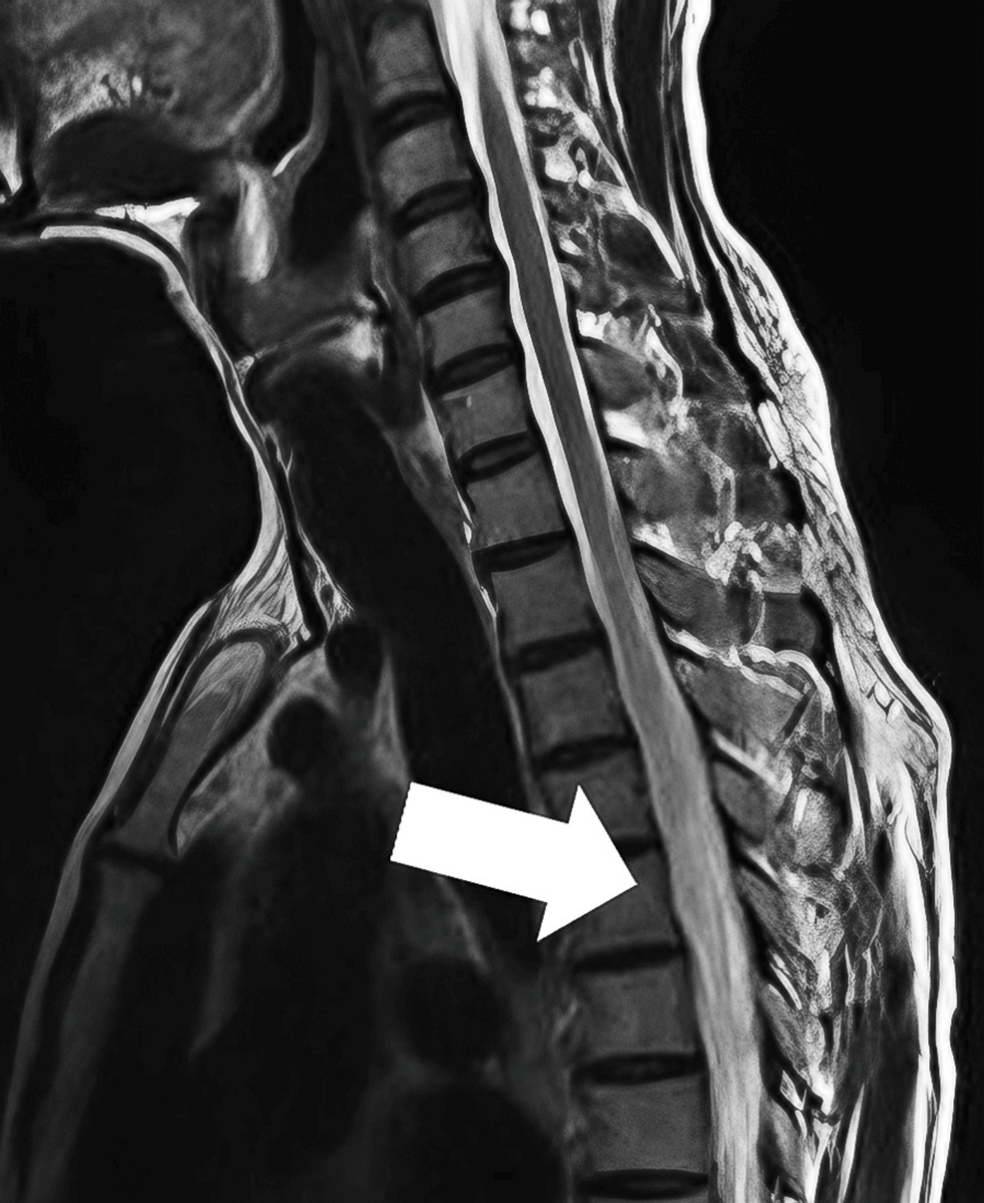
The spinal MRI. The MRI revealed an intramedullary T2 hyperintense signal abnormality in the central gray matter from the C5 to T4 levels of the spinal cord (as shown by the arrow). MRI: magnetic resonance imaging

MRI of the brain, orbits, and spine with IV contrast provided critical diagnostic insights. The brain and orbits showed no significant abnormalities, focusing concerns on the spinal cord. The spinal MRI revealed an intramedullary T2 hyperintense signal abnormality in the central gray matter from the C5 to T4 levels of the spinal cord, spanning approximately six vertebral segments of 4.5 cm (Figure 1). This lesion exhibited patchy areas of contrast enhancement and associated cord expansion and edema, consistent with active inflammation. These findings are characteristic of a longitudinally extensive TM lesion, confirming the diagnosis in the context of the clinical presentation. The MRI findings pointed to acute TM, with no evidence of intracranial or orbital involvement.

Upon presentation, differential diagnoses considered included acute flaccid myelitis, Guillain-Barré syndrome, and spinal cord compression due to various etiologies such as tumors or abscesses. The diagnostic process was guided by the patient’s clinical presentation, laboratory findings, and crucial imaging results. The MRI findings were pivotal, showing a longitudinally extensive TM lesion, which, coupled with the absence of bacterial growth in blood cultures and the specific pattern of urinary symptoms without significant infection, solidified TM as the definitive diagnosis.

Following the confirmation of TM, the patient was promptly started on disease-specific treatment, which included high-dose IV corticosteroids to reduce spinal cord inflammation and vancomycin as a precautionary measure against potential bacterial superinfections. Despite these initial treatments, the patient showed insufficient improvement, prompting the use of therapeutic plasmapheresis. After five days of corticosteroids, plasmapheresis was initiated, and three sessions were completed. This intervention led to a marked improvement in the patient’s condition.

The patient is currently on maintenance doses of steroids and antibiotics with close monitoring to manage any potential flare-ups or complications. The therapeutic response has been satisfactory, with significant recovery in motor functions and partial resolution of urinary and fecal incontinence. Long-term follow-up includes regular neurological assessments and potential adjustments in therapy based on the patient’s progress and symptomatology.

## Discussion

TM is a rare inflammatory disorder of the spinal cord, with an estimated annual incidence of 1-8 cases per million population, emphasizing the uncommon nature of this case. TM can affect individuals of any age, but it is relatively uncommon in children, accounting for about 25% of occurrences [3]. The clinical presentation in our patient included bilateral lower limb paralysis, sensory abnormalities, and autonomic dysfunction resulting in urinary and fecal incontinence, which are classic symptoms of TM [4]. An atypical feature in this case was the presence of high-grade fever at onset, suggesting a possible underlying infectious or post-infectious etiology.

Laboratory findings in our patient revealed mild anemia, elevated eosinophils, slightly low potassium levels, and mildly elevated ESR and CRP, indicating an inflammatory process. The MRI findings were crucial, showing a longitudinally extensive T2 hyperintense lesion in the spinal cord from the C5 to T4 levels with patchy areas of contrast enhancement, consistent with active inflammation. The patient’s insufficient response to high-dose IV corticosteroids led to the use of plasmapheresis, which resulted in significant clinical improvement. This case highlights the importance of considering aggressive therapeutic strategies in pediatric TM, especially in cases unresponsive to initial treatments, and underscores the critical role of early and accurate diagnosis combined with comprehensive diagnostic evaluations.

High-grade fever at the outset, on the other hand, is unusual for TM and could point to an underlying infectious or post-infectious etiology, which is not usually linked to TM in pediatric instances. The MRI results showed a longitudinally extensive lesion that extended across several spinal segments, from C5 to T4, with cord enlargement and contrast enhancement, typical of TM, but indicating a more severe presentation because of the lesion’s length (4.5 cm) [4]. This instance is further distinguished from other demyelinating illnesses, such as acute disseminated encephalomyelitis (ADEM) or NMOSD, by the lack of notable intracranial or orbital abnormalities.

Our initial treatment strategy of administering high-dose IV corticosteroids followed by plasmapheresis reflects the standard management protocols for acute TM, particularly in cases refractory to corticosteroids alone [5]. The response to treatment was satisfactory, though not a complete resolution, which aligns with the variable prognosis often reported in TM cases, where some patients continue to experience residual neurological deficits [6]. Alternative treatment options for TM include IV immunoglobulin (IVIG) and cyclophosphamide, which can be considered in severe or refractory cases. IVIG has been used with some success in reducing inflammation and promoting recovery, while cyclophosphamide is typically reserved for severe cases due to its potent immunosuppressive effects and associated risks. Exploring these alternatives can provide additional avenues for improving outcomes in pediatric TM.

The results of TM can be considerably improved with early intervention using corticosteroids and plasmapheresis, especially in terms of lowering the length and degree of disability, according to a review of the literature. The initial line of treatment frequently uses corticosteroids because of their strong anti-inflammatory properties, which help to lessen spinal cord swelling and inflammation. One example of this is high-dose IV methylprednisolone. In individuals with TM, its early use has been linked to better functional recovery and fewer neurological impairments. Plasmapheresis is regarded as an additional therapy when patients do not respond well to corticosteroids. The mechanism of action of plasmapheresis is the elimination of immune complexes and circulating antibodies that can exacerbate TM’s inflammatory process. According to a number of studies, plasmapheresis can be especially helpful in cases of TM that are severe or resistant to corticosteroid treatment when corticosteroid treatment alone is insufficient. This can improve the overall efficacy of treatment [7].

Additionally, prognosis guidance and treatment intensity customization may be achieved by early MRI and monitoring of inflammatory markers, according to recent studies. Erythrocyte sedimentation rate (ESR) and CRP levels are two examples of inflammatory markers that can be used to track an ongoing inflammatory process and a patient’s reaction to treatment. Increased levels of these markers might be a sign of ongoing inflammation, requiring more intensive care. Early MRI is essential for the diagnosis, assessment, and treatment of TM since it can show the degree of spinal cord swelling, the location and size of spinal cord lesions, and the presence of contrast enhancement. Given that longitudinally large lesions spanning numerous vertebral segments are frequently linked to a more severe disease course and worse outcomes, our findings offer important prognostic information. A tailored approach to treatment is made possible by the integration of clinical, laboratory, and imaging data, which also optimizes therapeutic approaches and improves patient outcomes [8].

This case report’s strength lies in its detailed clinical presentation, comprehensive diagnostic evaluation, and timely initiation of appropriate therapies, which together contribute to the depth of understanding of TM in pediatric cases. However, limitations include the absence of a longer follow-up period, which could provide more insights into the long-term outcomes and effectiveness of the interventions. Additionally, not exploring a broader spectrum of autoimmune markers might have limited our understanding of potential underlying etiologies.

This case provides several novel insights that enhance the educational value of both clinical practice and medical education, particularly in the realm of pediatric neurology. First, the atypical presentation of high-grade fever in TM highlights the need for clinicians to consider a broader differential diagnosis when faced with neurological symptoms accompanied by systemic signs. This case illustrates the importance of considering both typical and atypical presentations in rare disorders, emphasizing that early diagnostic imaging and comprehensive testing can significantly impact clinical outcomes. Additionally, the successful use of plasmapheresis after corticosteroids did not yield the desired effect, so it offers a practical example of escalating therapy based on patient response, which is a critical aspect of personalized medicine. This case also serves to remind healthcare providers of the potential severity of TM in children, underscoring the need for swift intervention to prevent lasting disability. Finally, the discussion of treatment strategies and outcomes in this case contributes to the growing body of literature that aids in refining guidelines for managing acute episodes of TM, particularly in pediatric patients. These insights not only reinforce existing knowledge but also push the boundaries of our current understanding of pediatric TM management.

## Conclusions

This case report details a rare presentation of TM in a 10-year-old male, emphasizing the complexity of its diagnosis and management in pediatric neurology. The inclusion of high-grade fever and the effective use of plasmapheresis following corticosteroids highlight the importance of an adaptable treatment approach in cases unresponsive to initial therapies. Clinicians should consider early diagnostic imaging and a comprehensive inflammatory workup to identify TM promptly. For treatment, initiating high-dose corticosteroids followed by plasmapheresis in refractory cases can lead to significant improvements. Early and aggressive intervention is crucial for better outcomes in pediatric TM.
